# Dental stem cell-derived extracellular vesicles transfer miR-330-5p to treat traumatic brain injury by regulating microglia polarization

**DOI:** 10.1038/s41368-022-00191-3

**Published:** 2022-09-05

**Authors:** Ye Li, Meng Sun, Xinxin Wang, Xiaoyu Cao, Na Li, Dandan Pei, Ang Li

**Affiliations:** 1 Key Laboratory of Shaanxi Province for Craniofacial Precision Medicine Research, College of Stomatology, Xi’an Jiaotong University, Xi’an, China; 2 State Key Laboratory Breeding Base of Basic Science of Stomatology (Hubei-MOST) & Key Laboratory of Oral Biomedicine Ministry of Education, School and Hospital of Stomatology, Wuhan University, Wuhan, China; 3 Department of Periodontology, College of Stomatology, Xi’an Jiaotong University, Xi’an, China

**Keywords:** Stem cells, Mesenchymal stem cells

## Abstract

Traumatic brain injury (TBI) contributes to the key causative elements of neurological deficits. However, no effective therapeutics have been developed yet. In our previous work, extracellular vesicles (EVs) secreted by stem cells from human exfoliated deciduous teeth (SHED) offered new insights as potential strategies for functional recovery of TBI. The current study aims to elucidate the mechanism of action, providing novel therapeutic targets for future clinical interventions. With the miRNA array performed and Real-time PCR validated, we revealed the crucial function of miR-330-5p transferred by SHED-derived EVs (SHED-EVs) in regulating microglia, the critical immune modulator in central nervous system. MiR-330-5p targeted Ehmt2 and mediated the transcription of CXCL14 to promote M2 microglia polarization and inhibit M1 polarization. Identified in our in vivo data, SHED-EVs and their effector miR-330-5p alleviated the secretion of inflammatory cytokines and resumed the motor functional recovery of TBI rats. In summary, by transferring miR-330-5p, SHED-EVs favored anti-inflammatory microglia polarization through Ehmt2 mediated CXCL14 transcription in treating traumatic brain injury.

## Introduction

Being considered a common causes of morbidity and mortality among populations, traumatic brain injury (TBI) has become a major contributor that burdens global health.^1,2^ Until now, although symptomatic interventions, such as surgery and drug administration play significant roles in the management of TBI, the overall efficacy in altering prognoses is still a matter of debate.^3,4^ Accelerated evidence has indicated that stem cell-based therapy shed new lights for the functional recovery of TBI.^5,6^ Among which, dental-derived mesenchymal stem cells (DSCs) brought promising perspectives owing to their innate neurogenic potentials.^7^ Researches suggested that DSCs contributed to functional recovery of TBI, mentally and physically.^8,9^ Of note, extracellular vesicle (EV) secreted by DSCs were regarded as the therapeutic effectors.^10^

EVs from donor cells interact with recipient cells and mediate cell-cell communications.^11^ With abundant cargos inside, EVs exhibited regulatory roles to their recipient cells. Based on our previous results, EVs secreted by stem cells from human exfoliated deciduous teeth (SHED) attenuated neuro-inflammation and recovered the impaired motor functions of TBI rats,^12^ leaving a mechanistic concern into interactions between SHED-derived EVs (SHED-EVs) and their target cells.

Microglia are considered as the critical players in neuro-inflammation resulted from TBI.^13,14^ There are two subtypes of activation in microglia, the inflammatory M1 phenotype and the anti-inflammatory M2 phenotype.^15^ By transferring microglia polarization from M1 to M2, SHED-EVs played significant roles in regulating neuro-inflammation after TBI.^12^ Compared with proteins and lipids, a specific class of nucleic acids, namely microRNAs (miRNAs), were remarkably identified as key cargos of stem cell-derived EVs in the treatment of diseases.^16,17^ Revealing the regulatory roles of critical miRNA inside SHED-EVs that effects would provide a new therapeutic target for the treatment of TBI. Here in the current study, we employed both in vitro and in vivo experiments to determine the key therapeutic effector miRNA in SHED-EVs and the molecular mechanism in the treatment of TBI, providing novel therapeutic targets for future clinical interventions of TBI and other related neurological diseases.

## Results

### SHED-EVs transferred miR-330-5p to activated microglia

**Fig. 1:**
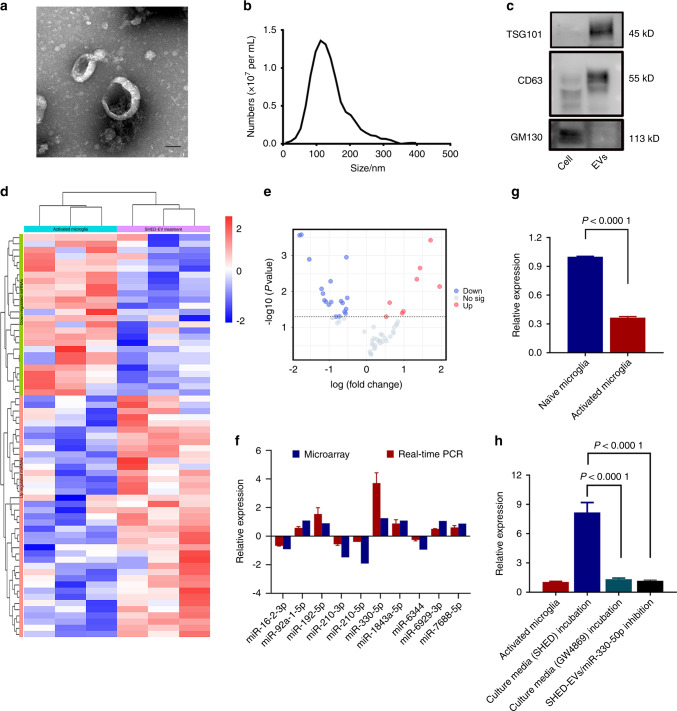
SHED-EVs transferred miR-330-5p to activated microglia. **a** Transmission electron micrographs of SHED-EVs. Scale bar: 50 nm. **b** Particle sizes measured by nanoparticle tracking analysis. **c** Western blotting characterization of EV markers. **d** Heatmap of differentially expressed miRNAs in activated microglia co-cultured with SHED-EVs. **e** Volcano plot filtering of differentially expressed miRNAs. **f** Real-time PCR validation of candidate miRNAs. **g** The level of miR-330-5p in activated microglia. **h** GW4869 inhibited the delivery of miR-330-5p from SHED-EVs to microglia. SHED-EVs without miR-330-5p may not alter the level of miR-330-5p in microglia

In our previous study, SHED-EVs contributed to functional recovery of rat TBI by shifting microglia M1/M2 polarization.^12^ To elucidate the molecular mechanisms underlying SHED-EV regulation, miRNA array was performed after EV characterization (Fig. 1a–c). We revealed 27 up-regulated miRNAs and 39 down-regulated miRNAs in activated microglia co-cultured with SHED-EVs (Fig. 1d). Further, the most highly ranked 10 miRNAs were selected that differentially expressed based on volcano plot filtering (Fig. 1e). The expression profile was confirmed in Fig. 1f. Accordingly, miR-330-5p was chosen as the potential effector based on the 4-fold increase in SHED-EV co-cultured microglia.

To quantitate the expression of miR-330-5p in naïve and activated microglia, we performed Real-Time PCR. Compared with naïve microglia, the expression of miR-330-5p was significantly inhibited in activated microglia (Fig. 1g). Consequently, we speculated that SHED-EVs transferred miR-330-5p to microglia to supplement the pathological loss induced by neuro-inflammation. To confirm the transfer of miR-330-5p by SHED-EVs, an EV inhibitor, GW4869, was added in the culture system of SHED. The collected culture media was used to treat activated microglia. Compared with microglia incubated with normal culture media of SHED, the expression of miR-330-5p was significantly reduced in the GW4869 group. We also specifically inhibited miR-330-5p in SHED with miR-330-5p inhibitor to obtain SHED-EVs without carrying miR-330-5p. Then the isolated SHED-EVs were incubated with activated microglia. As shown in Fig. 1h, with specific inhibition of miR-330-5p, SHED-EVs were not able to change the inner expression of miR-330-5p in the microglia, demonstrating the up-regulated miR-330-5p in microglia were mainly from SHED-EV transferring. In addition, the effect of SHED-EVs on microglial polarization under the specific inhibition of miR-330-5p was evaluated. As indicated in updated Fig. S1, without miR-330-5p contained, SHED-EVs may not regulate microglial polarization. Taken together, we revealed that SHED-EVs transferred miR-330-5p to activated microglia and may participate in microglia regulation in neuro-inflammation.

### miR-330-5p inhibited pro-inflammatory polarization and promoted anti-inflammatory polarization of microglia

**Fig. 2:**
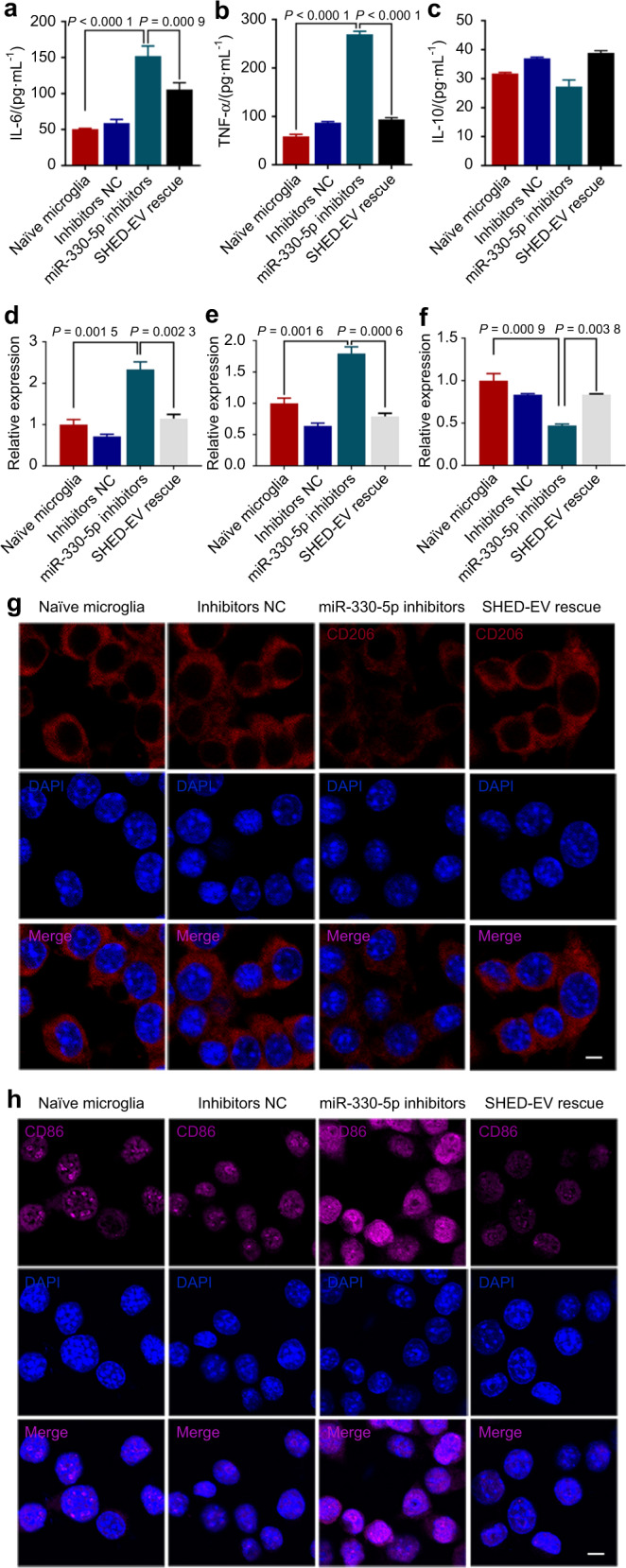
miR-330-5p inhibited pro-inflammatory polarization and promoted anti-inflammatory polarization of microglia. **a**–**c** The level of IL-6, TNF-α and IL-10 secreted by microglia. **d** Real-time PCR assay of M1 polarization marker CD86 mRNAs in microglia. **e** Real-time PCR assay of M1 polarization marker MHCII mRNAs in microglia. **f** Real-time PCR assay of M2 polarization marker IL-10 mRNAs in microglia. **g**, **h** Immunofluorescent staining for CD86 and CD206. Scale bar: 25 μm

To clarify the critical role of endogenous and exogenous miR-330-5p in regulating microglia, we inhibited miR-330-5p in naïve microglia (Fig. S2a) with the inhibitors and rescued miR-330-5p with SHED-EVs. As shown in Fig. 2a–c, the secretion of pro-inflammatory cytokines was increased when miR-330-5p was inhibited and SHED-EVs significantly reversed the effect. Nitrite concentration was determined by Griess assay. Increased nitrite concentration was found in the culture system of naïve microglia co-cultured with miR-330-5p inhibitors, while with the supplement of SHED-EVs, the level of nitrite was significantly inhibited (Fig. S2b). As indicated, miR-330-5p inhibition induced the up-regulated level of M1 polarization, while the level of M2 phenotype marker was down-regulated. SHED-EV rescue inhibited the pro-inflammatory M1 microglia polarization and induced the anti-inflammatory M2 polarization (Fig. 2d–h and Fig. S2c, d). Collectively, miR-330-5p played a critical role in the regulation of microglial polarization.

### Ehmt2 was the downstream target of miR-330-5p

**Fig. 3:**
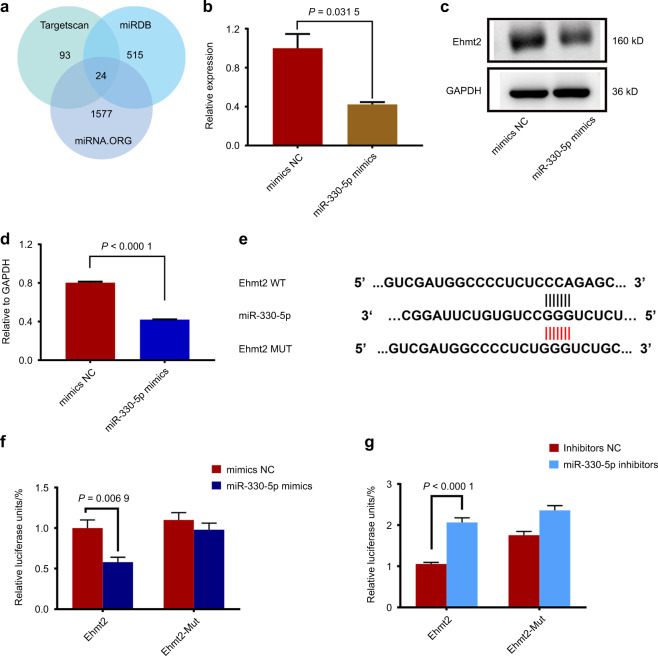
Ehmt2 was the downstream target of miR-330-5p. **a** Target prediction of miR-330-5p. **b** The mRNA level of Ehmt2 regulated by miR-330-5p. **c** The protein level of Ehmt2. **d** Western Blotting quantification. **e** Luciferase reporter designing. **f**, **g** Transfection of miR-330-5p mimics or inhibitors with luciferase reporter

To explore the mechanism of miR-330-5p in regulating microglial polarization, we predicted the potential targets from three databases (Targetscan, microRNA.ORG, and miRDB). Since human-derived miR-330-5p has exactly the same structure as mouse-derived one, we employed mmu- miR-330-5p to predict the downstream targets (Fig. 3a). We further selected Ehmt2 from the 24 overlapped results for its association with microglia development.^18^ Real-time PCR was used for validation. The mRNA level of Ehmt2 was significantly inhibited by miR-330-5p (Fig. 3b). Western blotting was performed for further confirmation (Fig. 3c, d), indicating the negative regulation of miR-330-5p on Ehmt2. Moreover, according to the predicted binding site, the wild-type or mutant plasmid of Ehmt2 was constructed (Fig. 3e) and co-transfected into 293 T cells. According to the luciferase reporter assay, miR-330-5p mimics resulted in the decreased activity (Fig. 3f), while miR-330-5p inhibitors led to the increased activity of Ehmt2 (Fig. 3g), indicating the direct binding of miR-330-5p to Ehmt2.

**Fig. 4:**
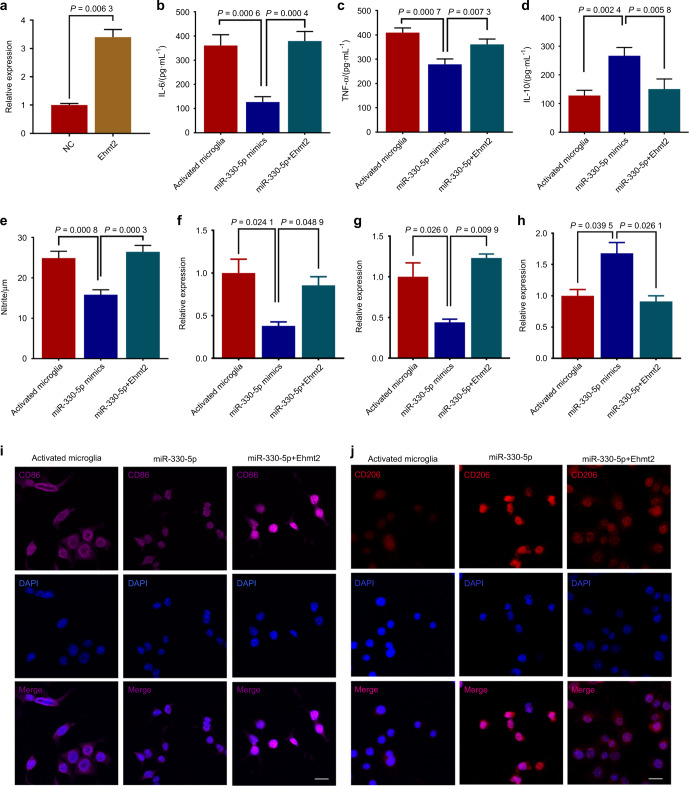
Ehmt2 reversed the effects of miR-330-5p on microglial polarization. **a** Ehmt2 overexpressed microglia construction. **b**–**d** The level of IL-6, TNF-α and IL-10 secreted by microglia. **e** NO levels quantified via Griess assay. **f** Real-time PCR assay of M1 polarization marker CD86 mRNAs in microglia. **g** Real-time PCR assay of M1 polarization marker MHCII mRNAs in microglia. **h** Real-time PCR assay of M2 polarization marker IL-10 mRNAs in microglia. **i**, **j** Immunofluorescent staining for CD86 and CD206. Scale bar: 100 μm

We further stably overexpressed Ehmt2 in BV-2 cells (Fig. 4a). As indicated in Fig. 4b–d, Ehmt2 significantly reversed the effect of miR-330-5p on microglial polarization. Specifically, Ehmt2 reversed the inhibition of pro-inflammatory cytokine secretion induced by miR-330-5p and abolished the promotion of anti-inflammatory cytokine. Moreover, Ehmt2 restored nitrite secreted by activated microglia (Fig. 4e). In addition, Ehmt2 resumed M1 polarization and erased M2 polarization (Fig. 4f–j and Fig. S3).

### miR-330-5p mediated the transcription of CXCL14 to regulate microglia polarization

**Fig. 5:**
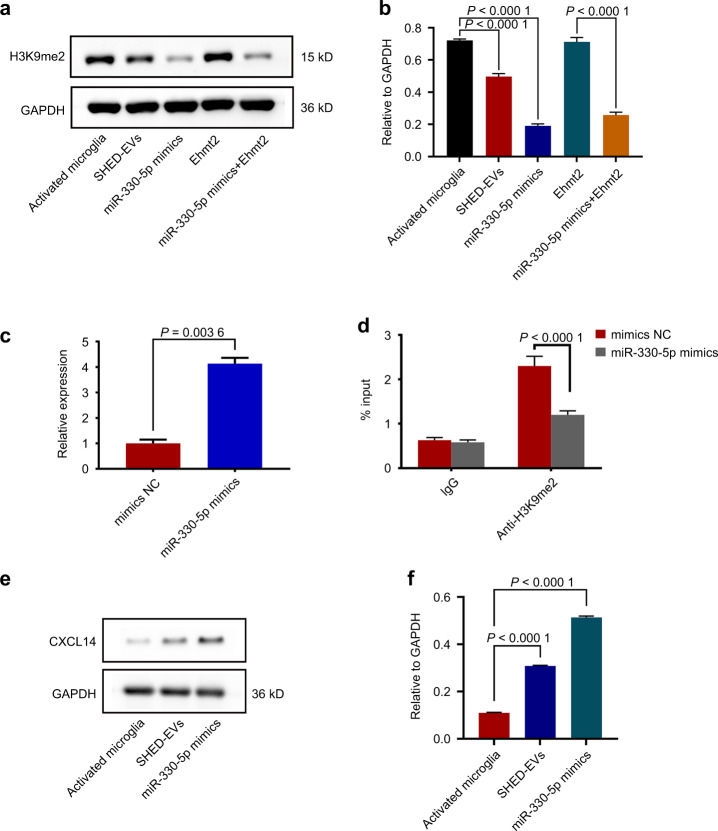
miR-330-5p mediated the transcription of CXCL14 to regulate microglia polarization. **a** Level of H3K9me2 in microglia regulated by SHED-EVs and miR-330-5p/Ehmt2. **b** Quantitative analysis. **c** Transcription level of CXCL14 in miR-330-5p overexpressed microglia. **d** The enrichment of H3K9me2 regulated by miR-330-5p. **e** Protein level of CXCL14 in microglia regulated by SHED-EVs and miR-330-5p. **f** Quantitative analysis

We further elucidated the signaling pathway mediated by miR-330-5p/Ehmt2 in microglia polarization. Ehmt2 involves in catalyzing the methylation of H3K9.^19^ To examine the role of miR-330-5p/Ehmt2 on H3K9me2, we evaluated the protein level of H3K9me2. As indicated in Fig. 5a, b, the level of H3K9me2 was significantly regulated by miR-330-5p/Ehmt2. H3K9me2 was reported to negatively regulate the transcription of CXCL14,^18^ the robust inducer of macrophage M2 polarization. As shown in Fig. 5c, miR-330-5p induced the accumulated transcription of CXCL14. It was further indicated that miR-330-5p inhibited the enrichment of H3K9me2 at the promoter region of CXCL14 (Fig. 5d), proving the role of miR-330-5p in the transcription of CXCL14. We further detected the protein level of CXCL14 that regulated by SHED-EVs/miR-330-5p. As indicated in Fig. 5e, f, CXCL14 was promoted by SHED-EVs/miR-330-5p.

### SHED-EVs/miR-330-5p ameliorated TBI in rats by regulating microglial polarization

**Fig. 6:**
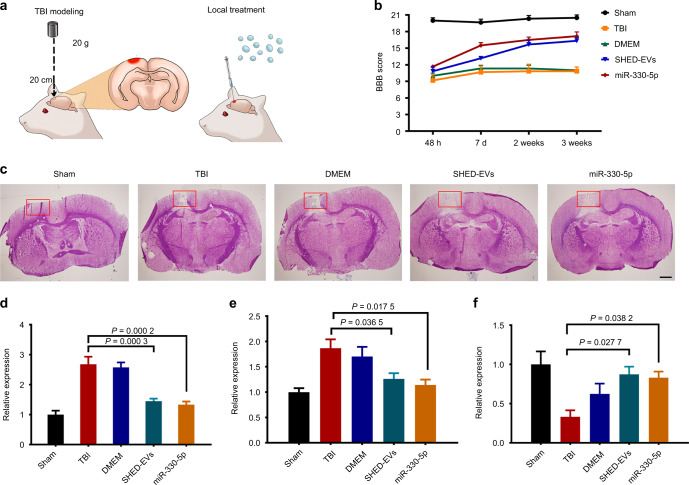
SHED-EVs/miR-330-5p ameliorated the functional damage of rat TBI. **a** Model construction and treatments. **b** Motor function analysis. **c** HE stained brain tissue slides. Scale bar: 200 μm. **d**–**f** The mRNA level of IL-6, TNF-α and IL-10 in brain tissues

**Fig. 7:**
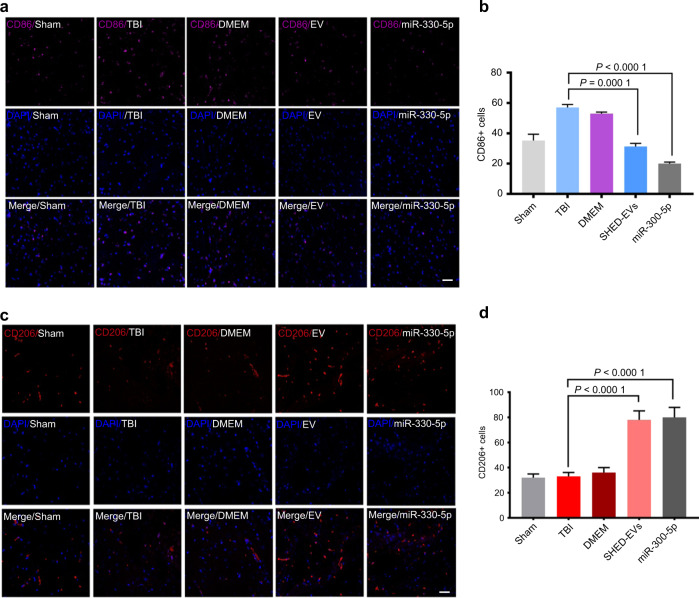
SHED-EVs/miR-330-5p shifted microglia polarization in brain tissues of TBI rats. **a**, **c** Immunofluorescent staining of brain tissues for CD86 and CD206. Scale bar: 200 μm. **b**, **d** Quantitative analysis

To determine the in vivo effect of SHED-EVs and their delivered miR-330-5p, we constructed the TBI model in rats and locally administrated SHED-EVs/miR-330-5p (Fig. 6a). Significant motor dysfunction was found at 48 h after TBI. To our expect, SHED-EVs/miR-330-5p promoted functional recovery of TBI rats at 7 days after injury (Fig. 6b). 2 weeks after SHED-EVs/miR-330-5p treatment, decreased lesion volume was shown in SHED-EVs/miR-330-5p group (Fig. 6c). As shown in Fig. 6d–f, down-regulated pro-inflammatory cytokines were detected in the SHED-EVs/miR-330-5p group, whereas up-regulated anti-inflammatory cytokine expression was found. Immunofluorescent staining was also performed to evaluated microglia polarization after SHED-EVs/miR-330-5p treatment. Demonstrated in Fig. 7 and Fig. S4, SHED-EVs/miR-330-5p shifted microglia polarization by regulating CXCL14 in TBI rats.

## Discussion

Over the past few years, EV-based stem cell therapies have been developed as promising solutions for neurological disorders. Of which, DSC-EVs draw particular attention for their embryonic neural crest origin.^20,21^ Comparing the PIWI-interacting RNAs in DSC-EVs and EVs from bone marrow mesenchymal stem cells, more neuronal information was found enriched in DSC-EVs.^22^ In this regard, it has also been suggested recently that DSC-EVs presented superior therapeutic potential in neurological diseases and thus may serve as better candidates for related therapy.

Our previous work has reported the phenotype of SHED-EVs in treating TBI through the regulation of microglia polarization, providing the basis for our mechanistic study in the current work. However, due to the restricted cell source and the limited production of SHED-EVs, the translation of SHED/ SHED-EVs in the treatment of TBI and related neurological disorders is hampered to a large extent. In this regard, the effective cargos in SHED-EVs should be clarified to not only expanded our knowledge on SHED-EVs, but also provide the solution for the shortage of SHED/ SHED-EVs for future translational medicine. With the better understanding of critical effectors in SHED-EVs and their mechanism of action, we may suggest potential interventions for TBI, facilitating TBI and related disease treatments. Among the cargos contained in EVs, miRNAs have been acknowledged to be pivotal in various neurological diseases and treatments. For instance, miR-7 from EVs targeted Parkinson Disease (PD) related gene SNCA that contributed to dopamine physiology and may involve in the increase of α-synuclein in PD patients.^23^ miR-216a-5p shuttled by MSC derived EVs could also repair spinal cord injury by regulating microglia.^24^ In our current work, miR-330-5p was considered as the key effector of SHED-EVs in the treatment of TBI. However, how miR-330-5p was sorted to SHED-EVs was largely unknown. Fully elucidation of the sorting mechanism would benefit effective TBI treatment with SHED-EVs in future translational medicine.

According to the current reports, the IL-4/IL-13 signaling pathway regulated by Stat6 was critical in microglial M2 polarization.^25^ Besides, the TGF-β1 signaling was also active during the process.^26^ Macrophages would shift to M2 phenotype when CREB-C/EBP-beta was enhanced.^27^ The Notch signaling generally considered as the critical regulator of development was also involved in macrophage polarization.^28^ NF-κB and MAPK activation was found in microglia M1/M2 polarization shifting.^29^ In the present study, we supplemented the mechanism of microglia polarization, also providing new potential targets for future TBI therapy.

Ehmt2 has been reported to participate in many pathophysiological processes of neurological disorders, especially Alzheimer’s disease.^30^ As indicated in our data, Ehmt2 regulated microglia polarization through H3K9me2 mediated transcription of CXCL14, a critical chemokine ligand in the central nervous system. H3K9me2 presented a negative role in gene expression since its recruitment of transcriptional repressors.^31^ CXCL14 is one of the best-known chemokines that regulated the integrity of hippocampal and maintained the balance in the adult brain.^32,33^ From the prospect of mechanism, miR-330-5p transferred by SHED-EVs would directly target Ehmt2 of microglia, inhibiting H3K9me2 and reducing the suppression of CXCL14. Taking the critical role of microglia polarization in Alzheimer’s disease into consideration, whether Ehmt2/H3K9me2/CXCL14 axis is also involved in the pathology of other neurological disorders should be discussed in the future study.

Cell-cell interactions are vital during stem cell therapy. Mainly through secretion of cytokines or transferring biological regulators through EVs, stem cell favored various diseases. Comparing to direct secretion, EVs may specifically target certain recipient cells to regulate their functions.^34^ Unveiling the potential mechanism behind SHED-EVs that targeting microglia would provide more valuable information in future translational medicine and would give more choices for enhancing the effect of SHED-EVs. In addition, for improving the therapeutic potential of SHED-EVs, engineering modifications, for example, miR-330-5p loading to SHED-EVs may be facilitated in future TBI interventions. Besides, future works could also concentrate on the basic knowledge of how the effective cargos (eg, miR-330-5p) are sorted into SHED-EVs to offer more valuable insights into the biological and functional hidden world of dental stem cells and their derived EVs.

## Conclusions

**Fig. 8:**
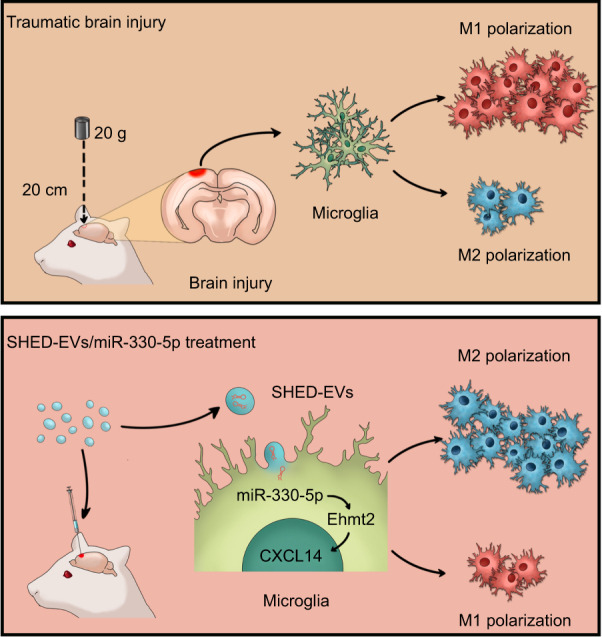
Mechanistic diagram of SHED-EVs/miR-330-5p in treating TBI. SHED-EVs/miR-330-5p shifted microglia polarization by targeting Ehmt2-H3K9me2 mediated CXCL14 transcription, leading to mitigated neuro-inflammation and enhanced repair of brain injury

To sum up, our current study revealed that miR-330-5p transferred from SHED-EVs rescued the impairment of motor function resulted from TBI. From the perspective of mechanistic, SHED-EVs/miR-330-5p shifted microglia polarization by targeting Ehmt2-H3K9me2 mediated CXCL14 transcription, leading to mitigated neuro-inflammation and enhanced repair of brain injury (Fig. 8).

## Materials and methods

### MiRNA microarray

EV isolation from the culture medium of SHED was performed as we reported previously.^12,35^ Detailed information was provided in the Supporting data. BV-2 microglia were activated by 1 μg·mL^−^^1^ lipopolysaccharide (LPS) for 24 h. Activated microglia were incubated with 50 μg·mL^−1^ SHED-EVs for 48 h. Both activated microglia and co-cultured microglia were obtained for miRNA extraction. miRNA microarray assay was performed and analyzed (Genechem, Shanghai, China).

### Real-Time PCR

Real-Time PCR was determined to validate the differential miRNA expression. Briefly, miRNA was extracted with RNAiso for Small RNA (9753Q, Takara Bio, Japan). Reverse transcription was performed by Mir-X miRNA First-Strand Synthesis Kit (638315, Takara Bio, Japan). The reverse-transcribed cDNA was detected by Mir-X miRNA qRT-PCR TB Green® Kit (638314, Takara Bio, Japan).

### ELISA, Griess assay and immunofluorescence

ELISA kit (KMC0062, BMS607HS, BMS614INST, Thermo Fisher, USA) was used to detect the secretion of IL-6, TNF-α and IL-10 secreted by microglia. Griess assay were performed with Griess Reagent Kit (G7921, Thermo Fisher, USA). Rabbit monoclonal to CD86 (ab234401, Abcam, UK) and Rabbit polyclonal to Mannose Receptor (ab64693, Abcam, UK) were used as primary antibodies. Goat Anti-Rabbit IgG H&L (A32733, Thermo Fisher Scientific, USA) and Goat Anti-Rabbit IgG H&L (ab150079, Abcam, UK) were used as secondary antibody. Detailed methods were provided in Supporting data.

### Target prediction of miR-330-5p and validation

TargetScan, miRDB and microRNA.ORG were employed for predicting potential targets of miR-330-5p. Ehmt2 was chosen from the intersection data for its critical role in regulating microglia. For target validation, a luciferase reporter assay was performed and detailed in Supporting data.

### Western blotting and ChIP-qPCR

Primary antibodies used in western blotting were Rabbit polyclonal to Ehmt2 (ab229455, Abcam, UK) and Rabbit monoclonal to Histone H3 (di methyl K9) (ab176882, Abcam, UK). ChIP-qPCR was performed as indicated in Supporting data.

### Construction of TBI rats and treatments

Thirty male Sprague-Dawley rats of about 200 g were used for TBI construction. Firstly, the cortical motor function area was drilled with a 2 mm circular window. Then the injured zone was vertically hit by a 20-g weight from 20 cm high. Injured rats were randomly assigned into five groups, *n* = 5.

For the Sham group: rats were only drilled without hitting; for the TBI group: TBI rats did not receive any treatments; for the DMEM group, TBI rats were locally injected with DMEM; for the SHED-EV group, TBI rats were treated with 200 μg SHED-EVs; for the miR-330-5p mimics group, TBI rats were injected with 2 μg miR-330-5p mimics. For functional studies, rats were tested within 21 days after treatments. For brain tissue staining, brain sections were prepared 48 h after treatments.

### Statistical analysis

Analyses between two groups and among different treatments were performed with Student’s *t* test and one-way ANOVA. *P* < 0.05 was considered as statistically significant.

## Supplementary information

supporting data

## Data Availability

The data that support the findings of this study are available from the corresponding author upon reasonable request.
