# Supplementary material for: Dental stem cell-derived extracellular vesicles transfer miR-330-5p to treat traumatic brain injury by regulating microglia polarization
Source: Int J Oral Sci. 2022 Sep 5;14:44. doi: 10.1038/s41368-022-00191-3 (PMC9445009; doi:10.1038/s41368-022-00191-3)
Supplement: Supplementary file 1 — supporting data [file 41368_2022_191_MOESM1_ESM.docx]

**Supplementary materials**

**Dental stem cell derived extracellular vesicles transferred miR-330-5p to regulate microglia polarization in the treatment of traumatic brain injury**

Ye Li^1,2^, Meng Sun^3^, Xinxin Wang^1^, Xiaoyu Cao^3^, Na Li^3^, Dandan Pei^1*^, Ang Li^1*^

^1^Key Laboratory of Shaanxi Province for Craniofacial Precision Medicine Research, College of Stomatology, Xi’an Jiaotong University, Xi’an, Shaanxi, China.

^2^The State Key Laboratory Breeding Base of Basic Science of Stomatology (Hubei-MOST) & Key Laboratory of Oral Biomedicine Ministry of Education, School and Hospital of Stomatology, Wuhan University, Wuhan, Hubei, China.

^3^Department of Periodontology, College of Stomatology, Xi’an Jiaotong University, Xi’an, Shaanxi, China.

**Correspondence:**

Ang Li and Dandan Pei

Key Laboratory of Shaanxi Province for Craniofacial Precision Medicine Research, College of Stomatology, Xi’an Jiaotong University, Xi Wu Lu No.98, Xi’an, Shaanxi, P. R. China.

+86 13002918118

E-mail: [drliang@mail.xjtu.edu.cn](mailto:drliang@mail.xjtu.edu.cn)

**Short title: Dental stem cell derived extracellular vesicles in the treatment of TBI**

**Materials and Methods**

*SHED-EV isolation*

SHED culture medium was centrifuged at 3000 × g for 15 minutes to remove cells and cell debris. Supernatant was collected to extract EVs with ExoQuick-TC. ExoQuick-TC and the supernatant were mixed before incubation overnight at 4°C. Then the mixture was centrifuged at 1500 × g at 4 ℃ for 30 minutes. EV pellet was resuspended in 50 µl sterile 1X PBS.

*ELISA*

Microglia were transfected with miR-330-5p inhibitors at 37 °C for 24 h. The concentration of IL-6, TNF-α and IL-10 in the culture medium was measured using ELISA kits according to the manufacturer’s instructions. Briefly, cell culture medium was added in the coated wells with IL-6, TNF-α and IL-10 mAb of 96-well and incubated for 2 hours at room temperature. The solution was thoroughly aspirated and washed 4 times with 1X Wash Buffer. Biotin conjugate solution was added into each well except the chromogen blanks and incubated for 30 minutes at room temperature. 1X Streptavidin-HRP solution was added for incubation at room temperature. After four times of washing, stabilized chromogen was added to each well and incubated for 30 minutes at room temperature in the dark. The absorbency was measured at 450nm by a microplate reader, and all samples were tested in triplicate.

*Griess assay*

An equal volume of culture medium of microglia, Griess Reagent and deionized water were mixed in each well of microplate and incubated for 30 minutes at room temperature. A mixture of 20 µL of Griess Reagent and 280 µL of deionized water was prepared as reference sample. The absorbance of the nitrite-containing samples relative to the reference sample was measured at 548 nm using a spectrophotometric microplate reader.

*Luciferase reporter assay*

293 T cells were cultured to approximately 70% confluence and then co-transfected with either wild type or mutant luciferase reporter vector and either miR-330-5p mimics/inhibitors or negative control by Lipofectamine® 3000. After 48 h transfection, the luciferase activity was measured using the Dual-Luciferase reporter assay kit (Promega, Madison, WI, USA). All experiments were conducted 3 times to ensure the reliability of the data.

*Western blotting*

The total protein was extracted using RIPA lysis buffer containing protease inhibitors (Beyotime, Shanghai, China). Total protein lysates were then centrifuged for 15 min at 12 000 × g at 4 °C. The lysates were mixed with loading buffer and denatured at 100 °C for 10 min. The products were then subjected to SDS polyacrylamide gel and transferred to polyvinylidene difluoride (PVDF) membranes (Millipore, Billerica, Massachusetts, USA). The membranes were blotted with 5% dehydrated milk for 2 h and then incubated with primary antibodies overnight. Secondary antibody incubation was performed for 2 h the next day followed by ECL(Millipore) exposure.

*ChIP-qPCR*

Cell was promptly fixed and crosslinked with 10% formaldehyde (The ratio of diluted formaldehyde to cell medium is 1:10.) for 10 minutes at room temperature with gentle shaking. Cross-linking was blocked by glycine in a proportion of 1:10 and incubated for 5 minutes at room temperature with gentle shaking. Then cells were washed in cold PBS and incubated with cold lysis buffer at 4℃ for 20 minutes. The mixture was centrifugated at 500 x g and 4°C for 5 minutes and the supernatant was discarded. The cell pellet was resuspended in 1 ml of ice-cold lysis buffer by pipetting up and down and incubated for 10 minutes at 4°C with gentle mixing on a rotator. The cells were pelleted again by centrifugation for 5 minutes at 500 x g and 4°C. And then the cell pellet was resuspended with protease inhibitor cocktail. The chromatin was sheared by sonication for 20 cycles. Then samples were centrifuged at 16,000 x g and 4°C for 10 minutes and the sheared chromatin was used for magnetic qPCR. 30 µg chromatins were incubated with antibodies against Ehmt2 or H3K9me2 overnight at 4°C. Next day, the beads were washed using 350μl of Wash Buffer W1, W2, W3 and W4, respectively. 2μl of diluted RNase cocktail was added to the aliquot of 50μl sheared chromatin and incubated for 1 hour at 37°C. Then the samples were incubated with 50μl of Elution Buffer E1, 4μl of Elution Buffer E2 at 65°C for 4 hours. DNA was purified with phenol-chloroform. Quantitative real-time PCR (RT-qPCR) was performed.

**Figures and legends**


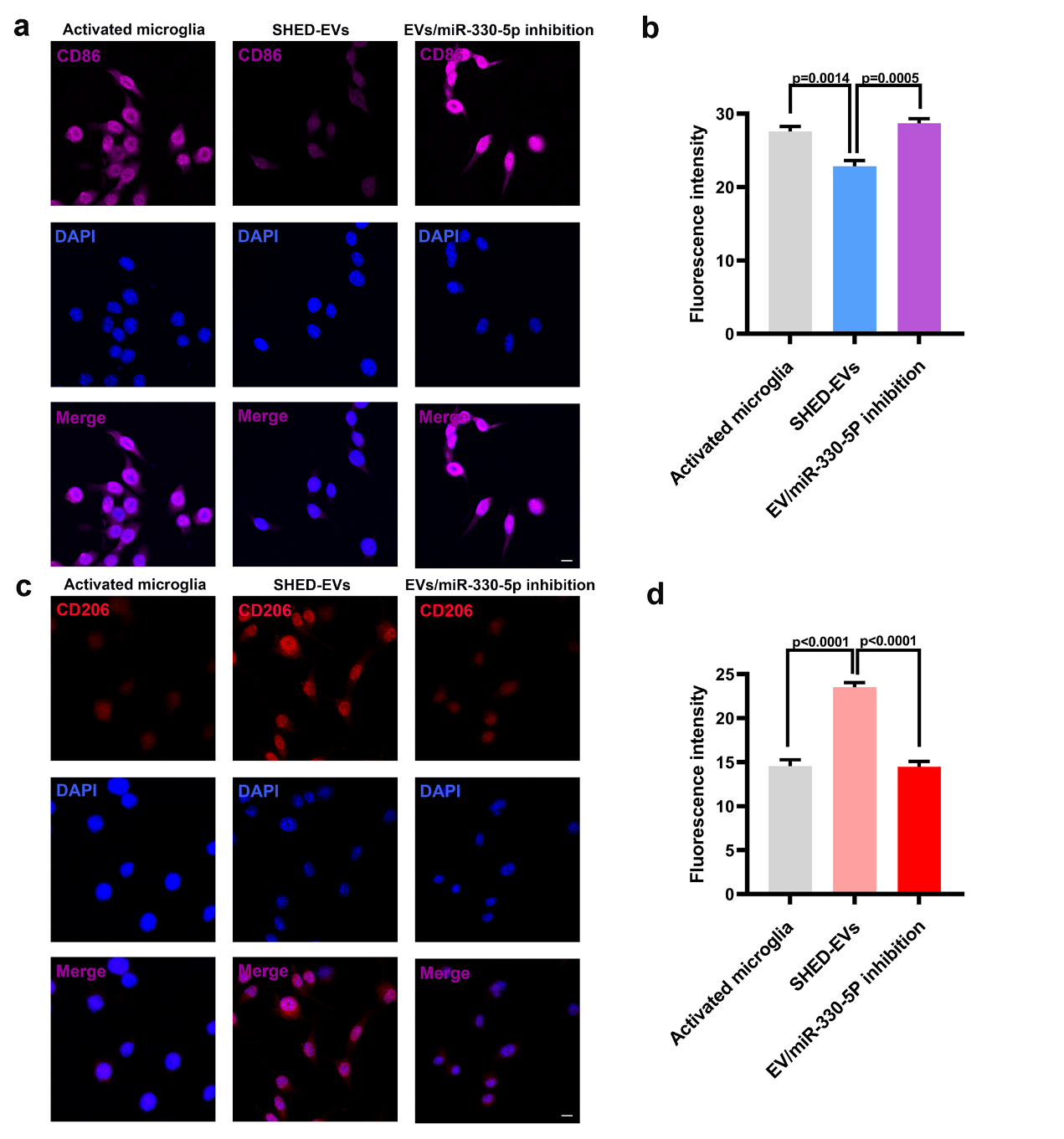


**Fig. S1 SHED-EVs shifted microglia polarization by transferring miR-330-5p.** (a, c) Immunofluorescent staining of microglia for CD86 and CD206. Scale bar: 50 μm. (b, d) Quantitative analysis.

**
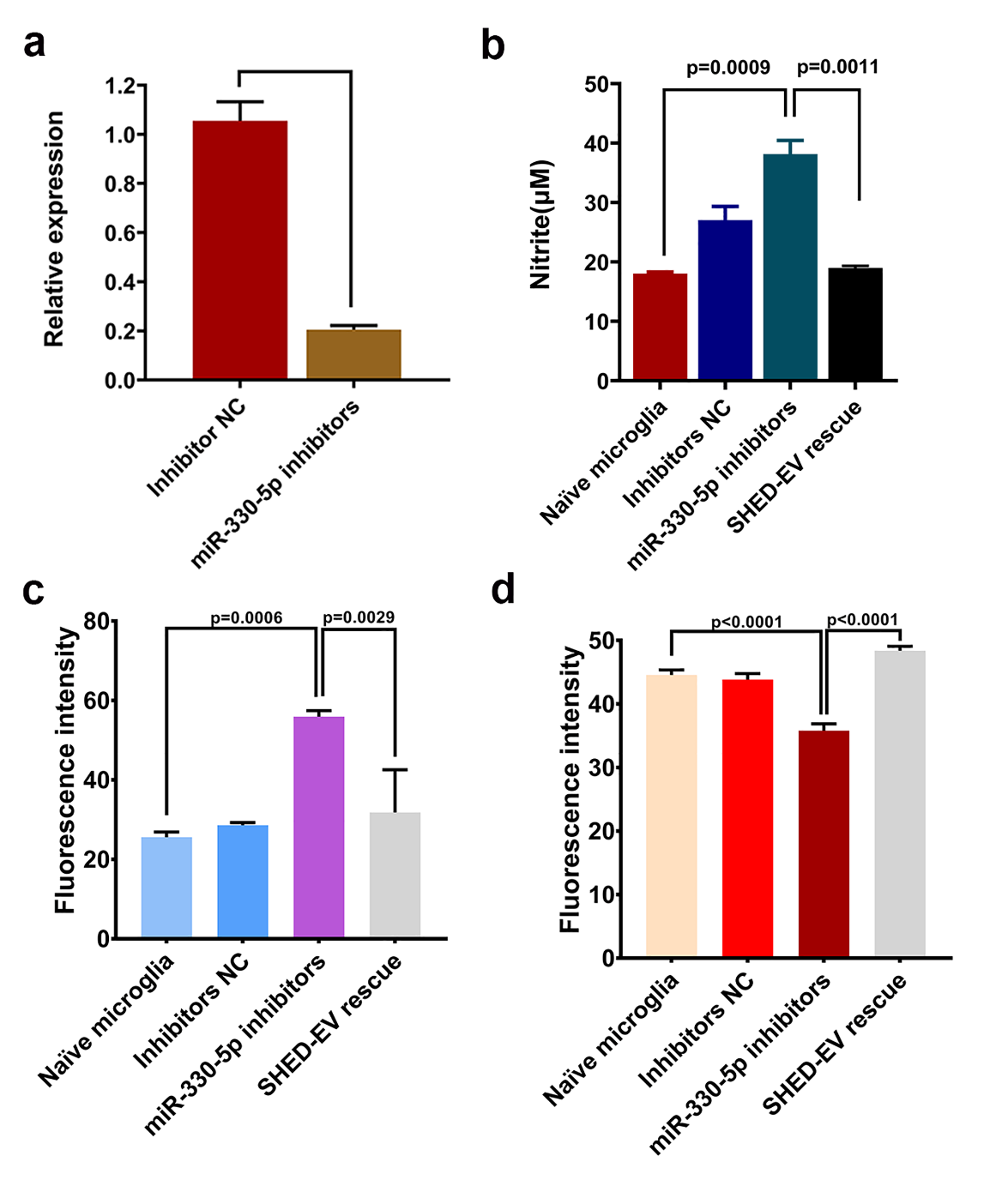
**

**Fig. S2 miR-330-5p inhibited pro-inflammatory polarization and promoted anti-inflammatory polarization of microglia.** (a) The efficiency of miR-330-5p inhibitors. (b) NO levels quantified via Griess assay. (c, d) Quantitative analysis of immunofluorescent staining.


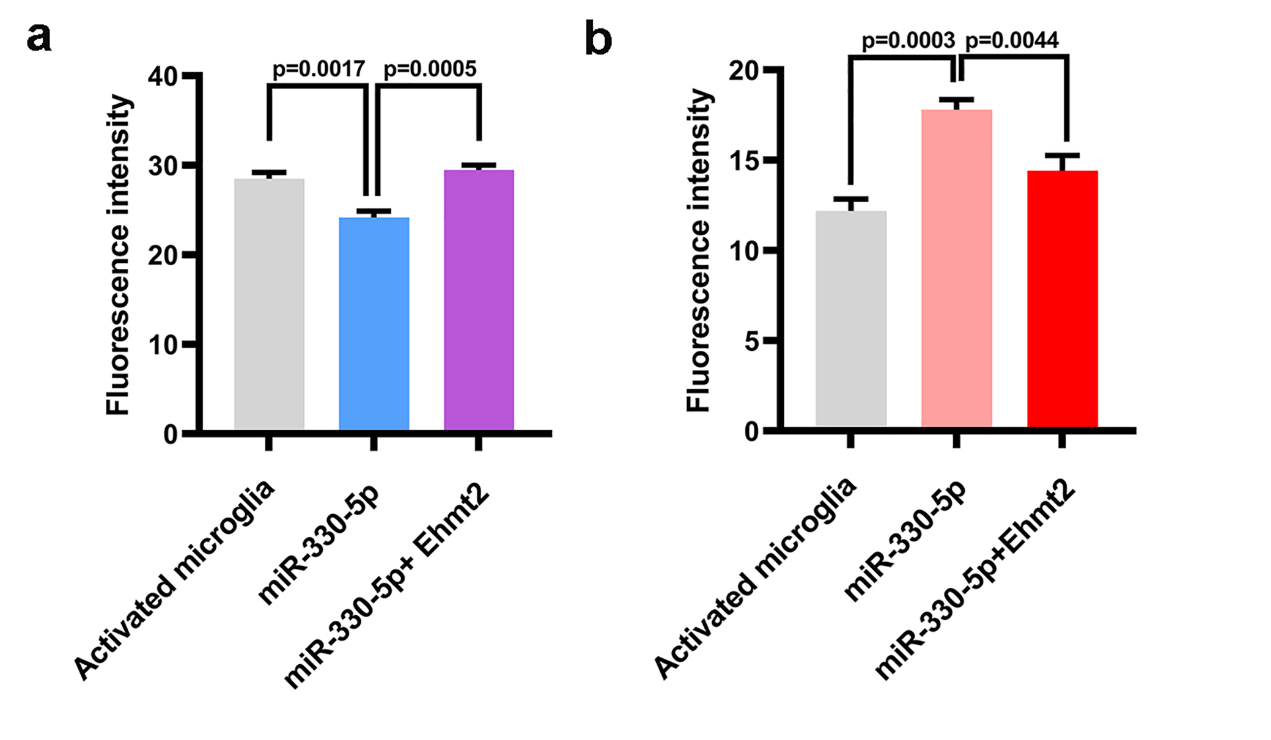


**Fig. S3 Ehmt2 reversed the effects of miR-330-5p on microglial polarization.** (a, b) Quantitative analysis of immunofluorescent staining for CD86 and CD206.


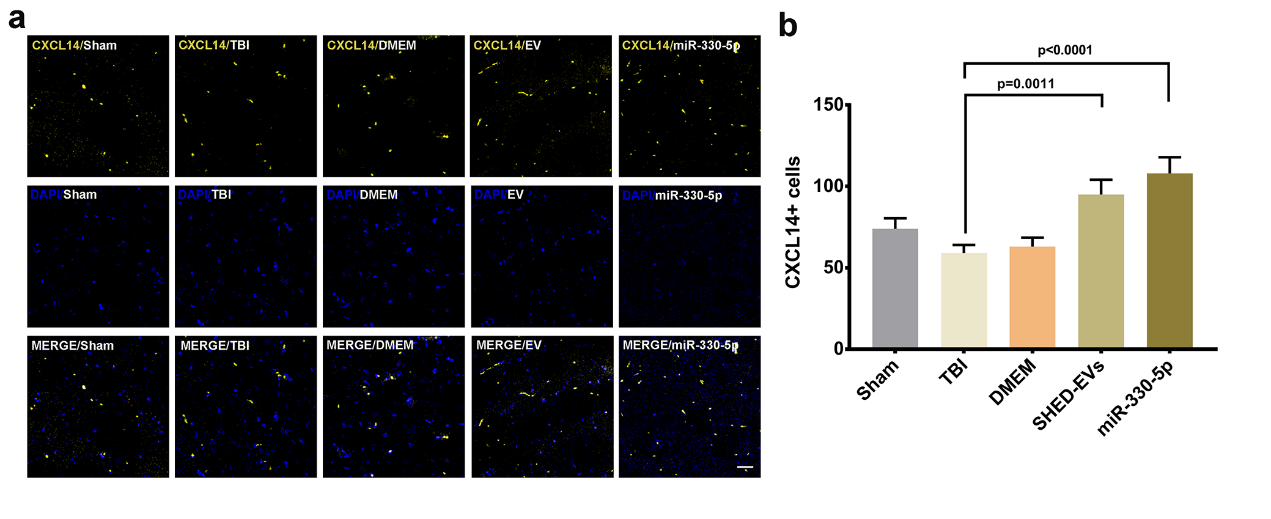


**Fig. S4 SHED-EVs/miR-330-5p shifted microglia polarization by regulating CXCL14 in brain tissues of TBI rats.** (a) Immunofluorescent staining of microglia for CXCL14 in brain tissues. Scale bar: 200 μm. (b) Quantitative analysis.
